# Systemic administration of all-trans retinoic acid modulated circulating immune cells but failed to improve healing in a preclinical model of musculoskeletal trauma

**DOI:** 10.3389/fimmu.2026.1683993

**Published:** 2026-07-28

**Authors:** Tyler Guyer, Dylan Gill, Kelly E. Leguineche, Kaitlyn Link, Kimberly A. Jones, Kylie E. Williams, Lia K. Strait, Kelly L. O’Neill, Molly Ann Hastings, Angela Lin, Drishti Maniar, Krishnendu Roy, Robert E. Guldberg

**Affiliations:** 1 University of Oregon, Eugene, OR, United States; 2 Wallace H. Coulter Department of Biomedical Engineering, Georgia Institute of Technology and Emory University, Atlanta, GA, United States; 3 Parker H. Petit Institute for Bioengineering and Bioscience, Georgia Institute of Technology, Atlanta, GA, United States; 4 Department of Biomedical Engineering, Vanderbilt University, Nashville, TN, United States; 5 Department of Pathology, Microbiology, and Immunology, Vanderbilt University Medical Center, Nashville, TN, United States; 6 Department of Chemical and Biomolecular Engineering, Vanderbilt University, Nashville, TN, United States

**Keywords:** ATRA, immune dysregulation, MDSCs, musculoskeletal trauma, non-union

## Abstract

Musculoskeletal trauma is exceedingly common and experiences a high incidence of complications, including bone non-union. Previous studies have implicated a dysregulated systemic immune response after injury as an important factor contributing to complications. We have previously identified a negative correlation between circulating myeloid-derived suppressor cells (MDSCs) and bone regeneration in a rat model of composite bone and muscle trauma. MDSCs are an immature, heterogeneous cell population of myeloid lineage that expands after injury and possesses potent immunosuppressive functions including the inhibition of T cells and expansion of regulatory T cells (Tregs), which could contribute to poor outcomes. As such, they may represent a novel therapeutic target in bone trauma. All-trans retinoic acid (ATRA) is a vitamin A derivative that has previously been shown to reduce MDSCs in cancer models by promoting their differentiation into mature myeloid cell populations. However, ATRA’s effects on MDSCs have not been explored in the context of trauma. Here, we investigated the effects of ATRA on peripheral blood immune cells both *in vitro* and *in vivo* using a rodent model of bone and muscle trauma. Treatment with ATRA depleted MDSCs in blood derived from traumatized rats *in vitro*. Further, *in vivo* studies revealed that a specific subset of MDSCs were depleted by systemic ATRA administration. Tregs were also depleted and correlated negatively with endpoint bone healing, although healing overall was not altered by ATRA treatment. Additionally, higher doses of ATRA had the unanticipated effect of increasing rather than decreasing circulating MDSC levels. Further studies will be necessary to develop and optimize immunomodulatory interventions targeting systemic immune dysregulation after trauma and to determine whether such interventions possess the potential to improve functional bone regenerative outcomes.

## 1 Introduction

Although recent advancements in care have improved survival for patients with severe musculoskeletal trauma, serious complication rates remain high, with non-union occurring in an estimated 1.9%–4.9% of all bone fractures (1, 2). Additionally, extensive concomitant soft tissue damage can further increase the risk of non-union. Patients with such complications are frequently faced with costly revision surgeries that prolong pain and may still result in permanent functional impairment (3, 4). As such, there remains a need for the development of novel therapeutic strategies that can reduce non-union incidence and alleviate the suffering associated with non-union.

Immune cells are well understood to play critical roles in bone fracture healing. During the early stage of inflammation, immune cells participate in angiogenesis and affect the recruitment, proliferation, differentiation, and activation of mesenchymal and hematopoietic cells at the injury site (5). While the research to date has primarily examined the effects of immune cells within the injury site, in recent years, systemic or circulating immune markers have also been implicated as potential factors in healing outcomes (6, 7). Following severe trauma, damage-associated molecular patterns and pathogen-associated molecular patterns are rapidly elevated and subsequently result in an increase in inflammatory cells and mediators (8). Left unchecked, excessive inflammation may lead to systemic tissue damage, resulting in multiple organ dysfunction syndrome and even death (9). Consequently, the elevation of inflammatory markers coincides with an increase in anti-inflammatory markers which act to balance systemic inflammation (10). Ideally, these concurrent inflammatory and anti-inflammatory responses would resolve within days and circulating levels of their corresponding immune mediators would return to baseline values. If this balance is not achieved, however, dysregulated levels of inflammatory and anti-inflammatory mediators can persist for days or weeks, which may contribute to impaired healing and systemic complications (11, 12).

A notable immune cell population that has recently been implicated in poor trauma outcomes such as non-unions is the myeloid-derived suppressor cell (MDSC). MDSCs represent a heterogeneous population of immature myeloid cells which expand rapidly following traumatic injury (13, 14). These cells have potent immunosuppressive functions, including inhibition of immune effector cells such as T cells and natural killer cells and induction or recruitment of other immunosuppressive cell types such as regulatory T cells (Tregs) (15–18). MDSCs exert these effects through mediators including reactive oxygen and nitrogen species, immunosuppressive cytokines such as interleukin-10 and TGF-beta, and enzymes such as arginase 1 (19–23). Various immune effector cell populations including certain subsets of T cells have been shown to be important to the early stages of bone repair (24), and therefore excessive immunosuppression could be detrimental to healing. In fact, recent studies have indicated a link between systemic levels of MDSCs and poor outcomes following bone trauma. In multiple rat models of musculoskeletal trauma, circulating MDSCs have been found to negatively correlate with bone regeneration (7, 25). In another study that aimed to target and deplete MDSCs in a rat trauma model using a nanoparticle-based immunomodulatory therapeutic, one sub-population of MDSCs decreased while another sub-population expanded, resulting in an increase in total MDSCs that correlated negatively with healing (26). Another study using a rat model of trauma associated with infection demonstrated an increase in circulating MDSCs along with decreased circulating T cells and a dysregulated cytokine response in rats with infection as compared to those with non-infected trauma (27). In humans, the relationship between MDSCs and trauma outcomes remains underexplored. However, in patients who develop sepsis, circulating MDSCs appear to be major actors of sepsis-induced immunosuppression and have been correlated with greater mortality and development of nosocomial infections (11, 28). Taken together, these animal and human studies may indicate potential for MDSCs as a therapeutic target for combating trauma-induced immunosuppression and improving healing outcomes.

Methods for therapeutically modulating MDSCs have been studied extensively in the context of cancer, where the immunosuppressive activity of MDSCs can contribute to tumor growth and lessen the potency of cancer interventions (29). Numerous therapeutics for diminishing MDSCs have been researched. Notably, all-trans retinoic acid (ATRA), a metabolite derived from dietary Vitamin A, has been shown to diminish MDSCs by promoting their differentiation into mature myeloid cell types (30, 31). ATRA upregulates the synthesis of glutathione, an oxygen scavenger, which in turn reduces levels of reactive oxygen species (32). Reactive oxygen species play roles in inhibiting MDSC differentiation (33), suggesting that reactive oxygen species scavenging compounds may rescue the differentiation trajectory into mature monocytes, granulocytes and dendritic cells. In certain cancer models, ATRA has been found to decrease the capacity of MDSCs to inhibit T cell function, yielding therapeutic benefits (34). However, its effects on MDSCs in the context of trauma have not been previously examined.

Here, we examined the effects of ATRA on circulating immune cells and functional bone healing in a rat model of musculoskeletal trauma. We hypothesized that systemic ATRA administration after injury would diminish circulating MDSCs and improve bone regeneration. To investigate this, we employed a previously established rat model of composite bone and muscle trauma involving both a critically-sized bone defect and a volumetric muscle injury (35). The addition of the muscle injury in this model has been shown to worsen bone regenerative outcomes and exacerbate immune dysfunction, making this an ideal testbed for immunotherapeutics. We began with an *in vitro* experiment screening the immunomodulatory potential of ATRA and several other treatments including vitamin E, anti-CXCR2 antibodies, and anti-TSPAN33 antibodies, all of which have been shown to diminish MDSCs or alter their immunosuppressive function (36–38). Peripheral blood immune cells derived from rats with bone and muscle trauma were cultured *in vitro* for 24 hours with each therapeutic and analyzed via flow cytometry. ATRA was the only therapeutic tested to successfully deplete MDSCs, and it was therefore investigated further in subsequent *in vivo* experiments. In these experiments, a group of rats with bone and muscle trauma received multiple intraperitoneal injections with ATRA in the early days after injury when MDSCs have been shown to be most elevated. The critically-sized bone defects (8mm) were also treated with bone morphogenetic protein-2 (BMP-2) at a low dose (2.5µg) which has previously been shown to result in a range of healing responses after 12 weeks of healing (39). Circulating immune cells were quantified after ATRA treatment via cell staining and flow cytometry, and bone healing was assessed via radiographs, micro-computed tomography (micro-CT) scans, and torsional testing of regenerated femurs. Finally, after observing only partial depletion of MDSCs following ATRA treatment, an additional *in vivo* experiment was performed to examine the effects of ATRA delivered at higher doses. Overall, the resulting data demonstrate a novel strategy for modulating the immune response to musculoskeletal trauma and highlight a need for deeper characterization of the post-trauma immune response and its relationship with healing.

## 2 Materials and methods

### 2.1 Nanofiber mesh preparation

Poly(ϵ-caprolactone) (PC-12 PURASORB, Corbion, USA) was dissolved at 15% (w/v) in dichloromethane (DCM) and dimethylformamide (both Sigma-Aldrich, USA) at a 3:2 ratio for approximately 16 hours under gentle agitation. The polymer solution was loaded into a 3 mL syringe (FORTUNA, USA) with a 32-gauge metal nozzle (McMaster-Carr, USA). A custom-based solution electrospinning system was used to create a polymer coating onto a 5 mm diameter cylindrical collector (McMaster-Carr). The collector was placed at a distance of 13 cm from the printer head, and a voltage of 20 kV and a pressure of 1 bar were applied. The mandrel movement was programmed with Mach3 software (Artsoft, USA). Then, a solder was used to melt through the scaffold by setting the solder tip to 400 °C, and programming solder movements with AeroBasic software (Aerotech, USA). Scaffolds were removed from the mandrel by spraying them with 70% ethanol to detach the polymer from the aluminum mandrel. Scaffolds were then gently perforated to yield 1-mm diameter perforations, which have been shown to benefit vascular invasion during the repair process (40). The resulting scaffolds were 13 mm in length, with alternating 3 and 2 openings of 1 mm on each row.

### 2.2 Alginate BMP-2 preparation

Alginate hydrogel constructs were prepared under sterile conditions inside a laminar flow hood. The constructs were made by first creating a 3% w/v alginate solution by dissolving arginine-glycine-aspartic acid-functionalized alginate (FMC BioPolymer) in sterile alpha-MEM (Corning). Recombinant BMP-2 (Pfizer) was reconstituted in a solution containing 0.1% rat serum albumin (Sigma-Aldrich) in sterile 4 mM hydrochloric acid, which was then mixed with the alginate solution to yield 2.5 μg BMP-2 per 150 μL of final solution. The solution was cross-linked in an excess of calcium sulfate by thorough mixing, allowed to gel at room temperature for 30 minutes, and then stored in sterile syringes at 4 °C before use in surgeries the next day.

### 2.3 Animal model

For all experiments, female Sprague Dawley rats (Charles River Laboratories) were used, which were 14 weeks old and weighed between 220 and 260 grams at the time of surgeries. Rats were housed in pairs in individually ventilated caging with a PVC tunnel and nylon bones for enrichment. Food and filtered water were provided *ad libitum*. All animals were allowed to acclimate in cages for at least one week after arrival before any procedures were performed. Following surgeries, animals were individually housed for two weeks for better monitoring of postoperative recovery. Animals were randomly allocated to treatment and control groups. At experimental endpoints, euthanasia was performed with either CO_2_ asphyxiation or exsanguination via cardiac puncture and was confirmed by secondary cervical dislocation.

Sample sizes for *in vivo* experiments were determined *a priori* based on bone volume data and immunological data from previous studies and cohorts. Sample sizes were calculated using power analyses with an 80% power of detection and a level of significance of 0.05 (G*Power software). Based on these analyses, it was determined that 6 animal subjects were required for each of the groups receiving composite bone and muscle injuries, and 4 animal subjects were required for the naive, uninjured group. An additional subject was added to each of the treatment groups to account for possible attrition.

### 2.4 Surgical procedures

All animal care and experimental procedures were approved by the University of Oregon IACUC (Protocol 20-10). Surgical procedures have been previously described (35). Anesthesia was induced and maintained via isoflurane (VetOne) inhalation for all animal procedures. For analgesia, all animals received a single subcutaneous injection of buprenorphine HCL (0.03 mg/kg, Hikma Pharmaceuticals) and a subcutaneous injection of sustained-release buprenorphine (0.65 mg/kg, Ethiqa) prior to surgical procedures. Animals also received 3 mL of Lactated Ringer’s solution (VetOne) subcutaneously prior to surgical procedures. Briefly, an anterolateral skin incision was made in the thigh, followed by separation of the overlaying muscles to reach the femur. A radiolucent polysulfone internal fixation plate was placed to stabilize the femur, and a critically sized 8 mm defect was created in the mid-diaphysis of the femur using an oscillating saw. Additionally, an 8 mm diameter full-thickness muscle defect was created in the adjacent quadricep using a biopsy punch. For all rats used in the *in vivo* MDSC depletion experiments, a 6-mm diameter poly-caprolactone nanofiber mesh was then placed within the femur defect around the bone ends, and alginate containing BMP-2 was injected via syringe into the perforations in the mesh. For rats used in the *in vitro* MDSC depletion experiments, the bone defects were left untreated. In both groups, the muscle defects were also left untreated. The muscle was then closed with Vicryl suture, and the skin was closed with a subcutaneous Monocryl suture layer and wound clips. Following surgery, the incision sites were cleaned with sterile saline, and a mixture of Metronidazole (Viona Pharmaceuticals) and New-Skin Liquid Bandage was applied. Animals were provided DietGel^®^ Boost (Clear H_2_O) and Nutri-Cal gel (Vetoquinol) for post-operative nutritional support. All animals were monitored for post-operative complications for at least three days following surgeries.

### 2.5 *In vitro* MDSC depletion experiments

For our *in vitro* experiments, blood was collected from three rats with composite bone and muscle trauma at day 7 post-injury via cardiac puncture. Approximately 3–5 mL of blood was collected from each rat into Vacutainer lithium-heparin-coated tubes (BD Biosciences). Red blood cells were lysed using RBC Lysis Buffer (eBioscience) according to the manufacturer’s instructions. Cells were then washed, counted, pooled, and resuspended in RPMI media supplemented with 10% FBS (Gibco) and 1% penicillin-streptomycin (Gibco) before being plated in a 96-well plate with 1,000,000 cells per well. For the treatment groups, ATRA, vitamin E, anti-CXCR2 antibodies, or anti-TSPAN33 antibodies were added to the respective wells. Another group was left untreated. The concentrations of each reagent used as well as their suppliers and product numbers are listed in **Supplementary Table 1**. The concentrations of ATRA selected for experiments here were similar to those that have previously been reported to modulate MDSCs *in vitro* (34, 41). Cells were incubated at 37 °C for 24 hours before being collected for subsequent cell characterization via fluorescent antibody staining and flow cytometry. Cell viability was also assessed prior to staining using a NucleoCounter NC-200 automated cell counter. This experiment was also repeated with ATRA using blood from three uninjured, naive animals.

### 2.6 *In vivo* MDSC depletion experiments

For *in vivo* experiments, ATRA was delivered to rats at a dose of 1 mg/kg body weight, 5 mg/kg body weight, or 10 mg/kg body weight. These doses were similar to those that have previously been used in other disease models in rodents (34, 42). For delivery of ATRA at the 1 mg/kg dose, ATRA was dissolved using olive oil (US Pharmacopeia) as the solvent. For delivery of ATRA at the higher doses, ATRA was dissolved in dimethyl sulfoxide (DMSO, US Pharmacopeia). ATRA solutions were administered via intraperitoneal injection. Blood was collected from rats after ATRA treatment via the ventral tail artery using Vacuette blood collection needles (Greiner Bio-One). For experiments involving rats with composite bone and muscle injuries, ATRA was administered between 3 and 7 days post-injury, and blood draws were performed at various timepoints including day 3 or 4, day 7, week 2, week 4, week 9, and week 12 post-injury. For experiments involving uninjured naive rats, only a single ATRA injection was performed, and blood was collected 12 hours later. Approximately 400 µL of blood was collected at each timepoint into Microtainer lithium-heparin coated tubes (BD Biosciences) for analysis via flow cytometry. Red blood cells were lysed using RBC Lysis Buffer (eBioscience) according to the manufacturer’s instructions. Cells were subsequently fixed using Flow Cytometry Fixation Buffer (R&D Systems) and resuspended in FACS buffer containing 2% fetal bovine serum in phosphate-buffered saline for subsequent cell characterization via fluorescent antibody staining and flow cytometry.

### 2.7 Immune characterization

For immune cell characterization in both the *in vitro* and *in vivo* experiments, cells were first incubated for 10 minutes at 4 °C with anti-rat CD32 to prevent non-specific antibody binding with Fc receptors. Next, cells were stained by incubation for 25 minutes at 4 °C with various surface antibodies before being washed with FACS buffer. Cells were then permeabilized using Cytofix/Cytoperm buffer (BD Biosciences) and washed with Perm/Wash buffer (BD Biosciences) according to the manufacturer’s instructions before staining with intracellular antibodies. A list of antibody suppliers, product numbers, and dilutions has been included in **Supplementary Table 2**. Flow cytometry was then performed using a BD Accuri C6 flow cytometer. Data were analyzed using FlowJo software with gates positioned based on fluorescence minus one controls allowing <1% noise. The immune cell populations analyzed included myeloid-derived suppressor cells (HIS48+CD11b/c+), monocytes (CD68+CD11b/c+), B cells (B220+), T cells (CD3+), cytotoxic T cells (CD3+CD8+), helper T cells (CD3+CD4+), and regulatory T cells (CD3+CD4+FOXP3+). The gating strategy used for identifying these populations is included in **Supplementary Figure 1**.

### 2.8 Radiography and micro-computed tomography

In our first *in vivo* experiment, longitudinal bone regeneration at weeks 4, 8, and 12 post-trauma was assessed both qualitatively and quantitatively via radiographic imaging and micro-computed tomography (micro-CT), respectively. Digital radiographs were captured at 40 kV with an exposure time of 7 seconds using an UltraFocus digital radiography system (Faxitron). Micro-CT was performed on femurs using a vivaCT80 (Scanco Medical) both *in vivo* at weeks 4 and 8 and *ex vivo* at week 12 post-injury. The volume of interest consisted of the central 6.5 mm of the 8 mm femoral defect. Scans were performed with a 55-kVp voltage and a 145-μA current, and the voxel size was set to 48μm for *in vivo* scans and 24μm for *ex vivo* scans. To identify newly regenerated bone, a threshold corresponding to 50% of native cortical bone density was applied. For *in vivo* micro-CT imaging, rats were anesthetized for a duration of no more than 40 minutes at each timepoint.

### 2.9 Biomechanical testing

Torsional testing to failure was also performed to quantitatively assess functional bone regeneration. Rats were sacrificed at week 12 post-injury and femurs were excised, wrapped in PBS-soaked gauze, and frozen for three days at -20 °C after micro-CT imaging. On the day of testing, femurs were thawed, their fixation plates were removed, and their native bone ends were potted in Wood’s metal (Alfa Aesar). The femurs were then tested to failure in torsion at a rotation rate of 3 degrees per second using the ElectroForce 3220 testing system (TA Instruments). Failure strength was identified as the peak torque within the first 90 degrees of rotation, and torsional stiffness was calculated as the slope of the linear region before failure in the torque-rotation plot.

### 2.10 Statistical analysis

All data are reported as mean ± SEM. Statistical significance was determined using either the t test or ANOVA with appropriate *post hoc* tests. Significance was determined by p values less than 0.05. All statistical calculations were performed using GraphPad Prism software (Version 10.4.2, La Jolla, CA, USA).

## 3 Results

### 3.1 Rat trauma-derived MDSCs are depleted *in vitro* following ATRA treatment

*In vitro* experiments were conducted using peripheral blood mononuclear cells derived from either our rat model of composite bone and muscle trauma or naive, uninjured rats. Blood samples were cultured for 24 hours either untreated or treated with all-trans retinoic acid (ATRA) at one of three concentrations, vitamin E, anti-CXCR2 antibodies, or anti-TSPAN33 antibodies.

**Figure 1:**
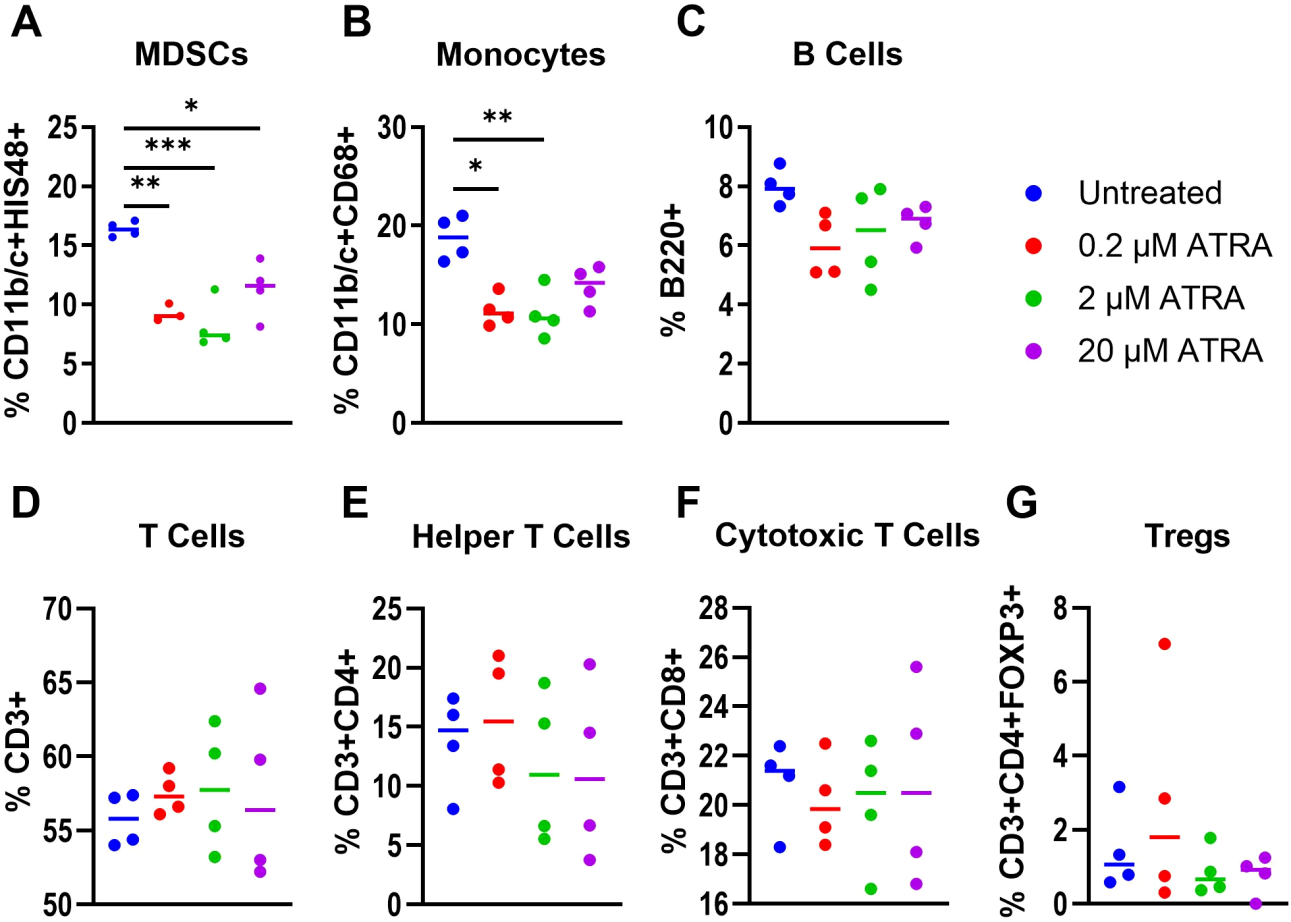
ATRA altered relative immune cell frequencies *in vitro* in blood derived from rats with composite bone and muscle trauma. **(A)** MDSCs were significantly depleted by treatment with ATRA at three different concentrations as compared to untreated controls. **(B)** Monocytes were significantly depleted by ATRA treatment at two of the concentrations tested. **(C–G)** Other immune cell populations were unaltered. Significance was determined using one-way ANOVA with Dunnett’s *post-hoc* test. *P < 0.05; **P < 0.01; ***P < 0.001 as indicated.

Treatment with ATRA at all three tested concentrations diminished myeloid-derived suppressor cells (MDSCs) in the trauma-derived blood as compared to untreated controls (**Figure 1A**). Monocytes were also significantly depleted in two of the three ATRA-treated groups (**Figure 1B**). Meanwhile, B cells, total T cells, helper T cells, cytotoxic T cells, and regulatory T cells (Tregs) were unaffected by ATRA treatment (**Figures 1C–G**). None of the other therapeutics tested *in vitro* resulted in altered blood MDSC levels, although anti-TSPAN33 antibodies altered the levels of B cells, monocytes, and T cells (**Supplementary Figure 2**). Lastly, in blood from uninjured animals, ATRA treatment was again shown to result in diminished MDSCs, although no other immune cell populations were significantly altered (**Supplementary Figure 3**). Total cell viability after the 24-hour culture was also found to remain consistent for all three ATRA concentrations tested *in vitro* (**Supplementary Figure 4**).

### 3.2 Circulating immune cells are altered *in vivo* following ATRA treatment

**Figure 2:**
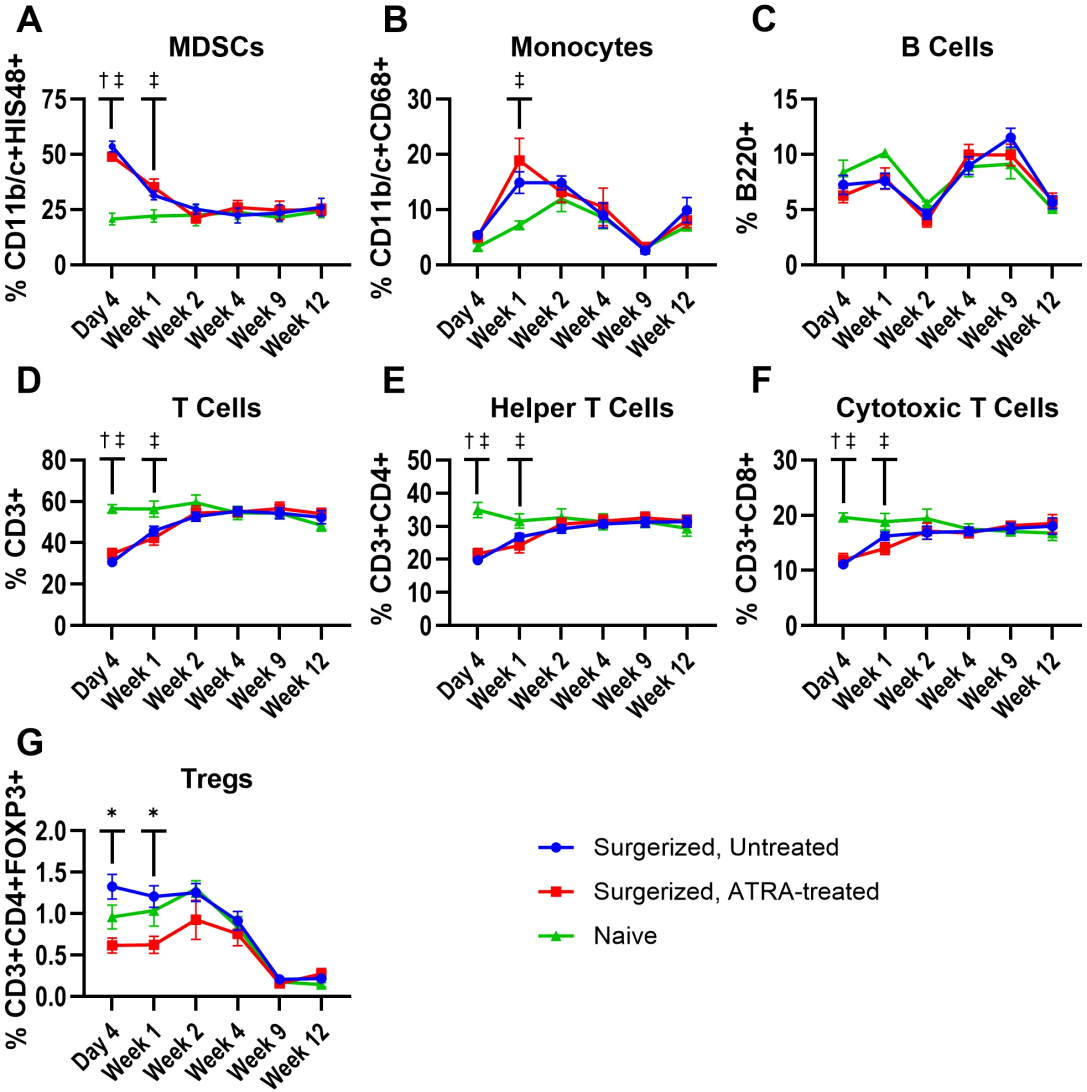
Peripheral blood immune cells were differentially regulated by trauma and by ATRA treatment *in vivo*. **(A)** MDSCs were elevated in rats with trauma as compared to uninjured rats at day 4 and week 1 post-injury. **(B)** Monocytes were also significantly elevated in one of the trauma groups at week 1 post-injury. **(D–F)** Total T cells, helper T cells, and cytotoxic T cells were diminished at day 4 and week 1 post-injury as compared to uninjured rats. **(G)** Tregs were significantly diminished following ATRA treatment in rats with trauma as compared to the untreated trauma group. Asterisks (*) indicate a significant difference between the ATRA-treated and untreated groups. Daggers (†) indicate a significant difference between the untreated and naive groups. Double daggers (‡) indicate a significant difference between the ATRA-treated and naive groups. n = 4 to 7 per group. Significance was determined using one-way ANOVA with Tukey’s *post-hoc* test. P<0.05 for all significant differences shown.

Given the results of the *in vitro* immunomodulatory experiments, ATRA was selected from the therapeutics tested for further study *in vivo* using our rat model of composite bone and muscle trauma. Here, a subset of injured rats received daily intraperitoneal injections with ATRA dissolved in olive oil at a dose of 1 mg/kg on days 3 through 7 post-injury. Blood was collected from rats in both the ATRA-treated and untreated groups at day 4, week 1, week 2, week 4, week 9, and week 12 after injury, and immune cells were characterized via flow cytometry. A naive control group consisting of age-matched uninjured rats was also included. The relative frequency of MDSCs in the blood was not significantly altered by ATRA treatment at any timepoint (**Figure 2A**). Tregs were significantly depleted in the ATRA-treated group at both day 4 and week 1 post-injury (**Figure 2G**). Compared to naive controls, both the ATRA-treated and untreated groups of injured rats had elevated MDSCs and decreased T cells, helper T cells, and cytotoxic T cells at day 4 and week 1 post-injury (**Figures 2A, B, D-F**).

**Figure 3:**
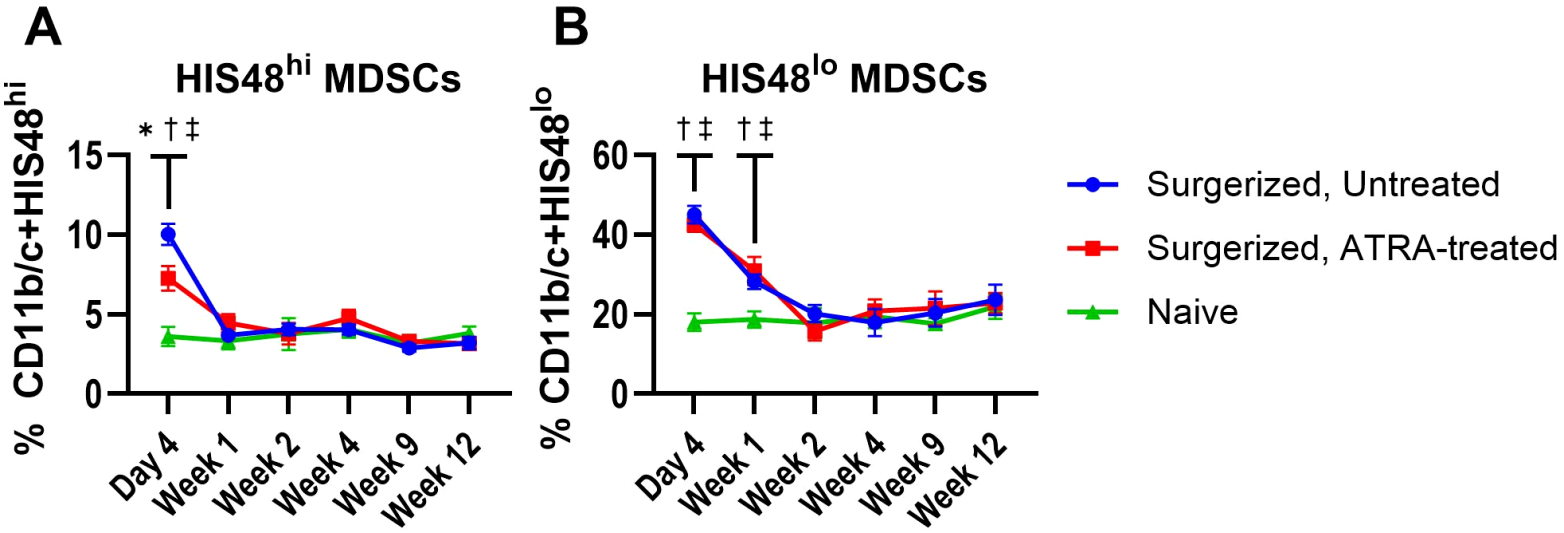
MDSC sub-populations were elevated by trauma and differentially regulated by ATRA treatment. **(A)** HIS48^hi^ MDSCs were elevated in both trauma groups as compared to the naive group and were diminished by ATRA treatment. **(B)** HIS48^lo^ MDSCs were also elevated in both trauma groups but were not diminished by ATRA treatment. Asterisks (*) indicate a significant difference between the ATRA-treated and untreated groups. Daggers (†) indicate a significant difference between the untreated and naive groups. Double daggers (‡) indicate a significant difference between the ATRA-treated and naive groups. n = 4 to 7 per group. Significance was determined using one-way ANOVA with Tukey’s *post-hoc* test. P<0.05 for all significant differences shown.

Additionally, a more extensive analysis of the MDSCs revealed that while total MDSCs were not depleted by ATRA treatment, a sub-population of the cells decreased. MDSCs possess two subsets in rats, which can be classified as CD11b/c^+^HIS48^hi^ and CD11b/c^+^HIS48^lo^. Here, the CD11b/c^+^HIS48^hi^ cells were depleted at day 4 post-injury (**Figure 3A**), while the CD11b/c^+^HIS48^lo^ cells were not altered (**Figure 3B**).

### 3.3 Treatment with ATRA did not alter functional bone regeneration

**Figure 4:**
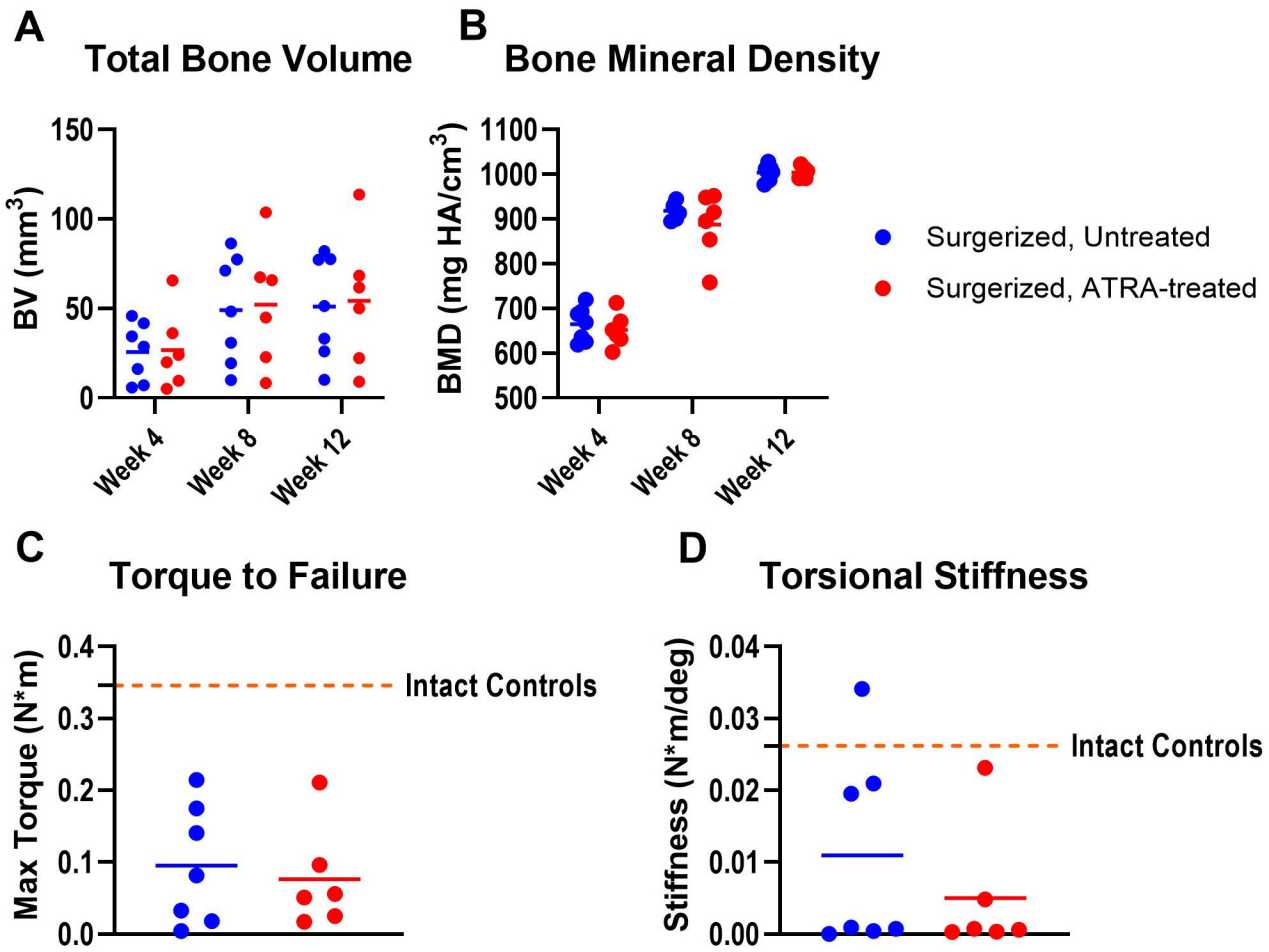
Functional bone regeneration was not significantly altered by treatment with ATRA. **(A, B)** Total bone volume **(A)** and bone mineral density **(B)** of regenerated bone as quantified by micro-CT. **(C, D)** Mechanical strength **(C)** and stiffness **(D)** of regenerated femurs as determined by *ex vivo* torsional testing to failure at week 12. Dotted lines indicate mechanical properties of intact femurs from the contralateral limbs of the trauma rats. Significance was assessed using a two-tailed *t* test for each comparison.

Bone healing was assessed by *in vivo* radiographic imaging and micro-computed tomography (micro-CT) at weeks 4 and 8 post-injury as well as *ex vivo* micro-CT and biomechanical testing at week 12 post-injury. Bone volume and bone mineral density were not significantly altered by ATRA treatment at any timepoint (**Figures 4A, B**). Similarly, endpoint torsional strength and torsional stiffness were not significantly altered (**Figures 4C, D**). Representative radiographs for both the treated and untreated trauma groups have been included in **Supplementary Figure 5**.

### 3.4 Circulating Tregs correlate with bone regeneration

**Figure 5:**
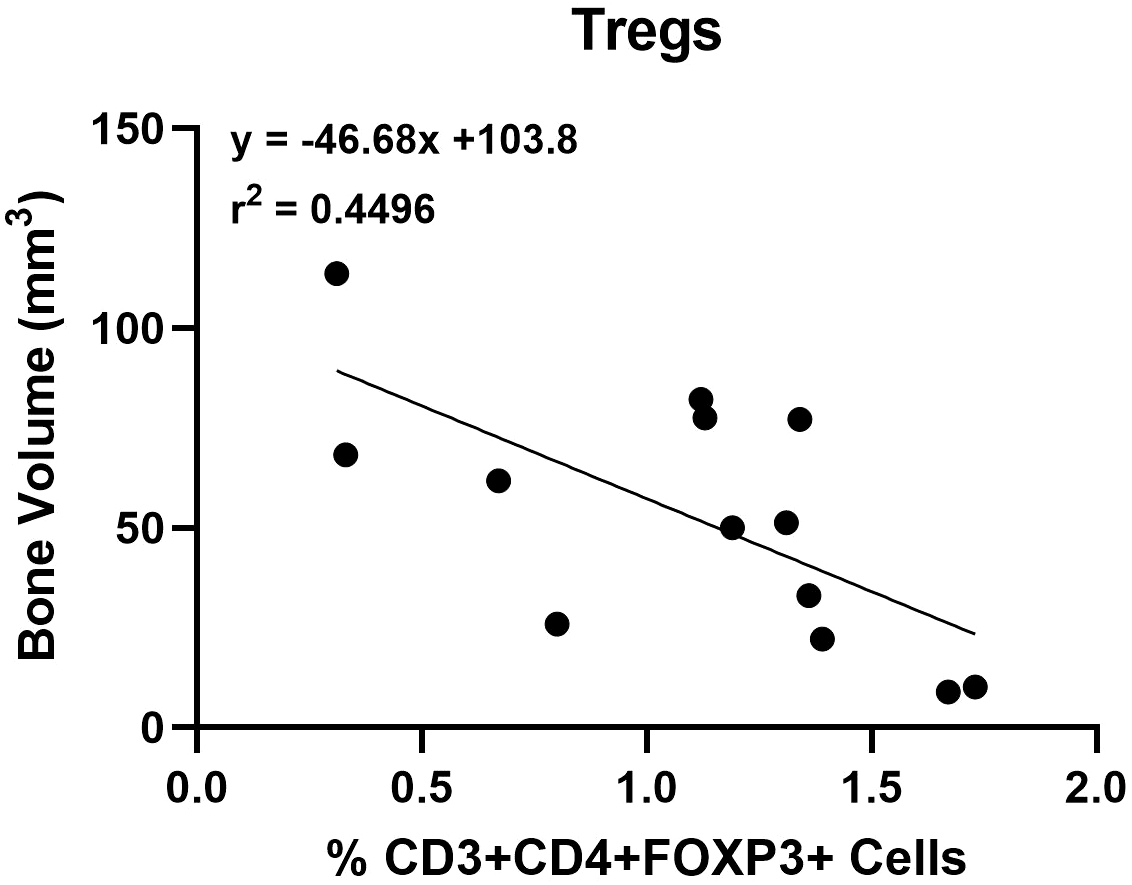
Tregs measured at two weeks post-injury correlate with bone regeneration. Week 2 Tregs correlated negatively with endpoint bone volume. Slope of linear regression significantly nonzero (P = 0.0121).

Linear regression analyses were performed using data from all rats in the trauma groups to identify systemic immune cell populations that correlated with bone regeneration. The relative frequency of circulating Tregs at week 2 post-injury correlated negatively with endpoint bone volume as measured by *ex vivo* micro-CT scans (**Figure 5**).

### 3.5 High dose ATRA increased circulating MDSCs in a rat model of trauma

**Figure 6:**
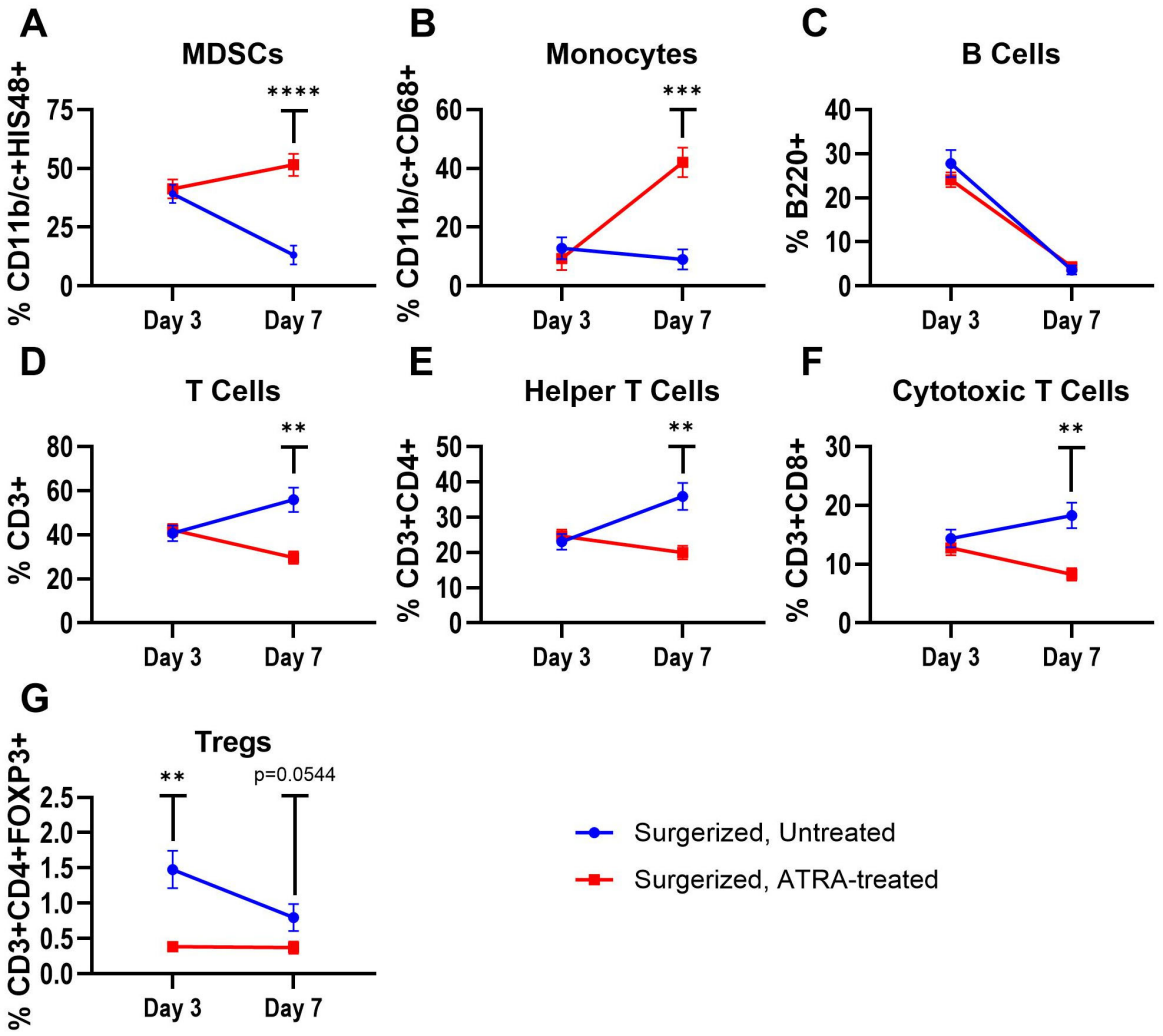
Treatment with ATRA at a higher dose resulted in elevated MDSCs. **(A)** MDSCs were not altered at day 3 following a single injection with ATRA at a dose of 5 mg/kg, but they unexpectedly increased following a second ATRA injection at day 7, as compared to untreated controls. **(B)** Monocytes were also elevated at day 7. **(D–F)** Relative frequencies of total T cells, helper T cells, and cytotoxic T cells decreased at day 7 in the ATRA-treated group as compared to the untreated control group. **(G)** Tregs were significantly diminished by ATRA treatment at day 3. n = 6 to 7 per group. Significance was determined using a two-tailed *t* test for each comparison. **P < 0.01; ***P < 0.001; ****P < 0.0001 as indicated.

Given that ATRA diminished a subset of MDSCs and Tregs in the previous *in vivo* study, a subsequent study was performed to determine whether ATRA at a higher dose could yield greater depletion of total MDSCs and modulate the healing response. Here, rats with the same bone and muscle injuries as in the previous study received intraperitoneal injections with ATRA at a dose of 5 mg/kg on days 3 and 7 post-injury. Notably, in this study, the higher ATRA dose necessitated the solvent DMSO rather than olive oil. Blood draws were performed 12 hours after each injection and immune cells were analyzed via flow cytometry. Total MDSCs were once again not altered following the first day of ATRA injections, while Tregs were again depleted (**Figures 6A, G**). Interestingly, however, MDSCs were increased on day 7 in the ATRA-treated group as compared to the untreated group following the second set of injections. Monocytes also increased significantly in the ATRA-treated group at this timepoint, while the relative frequencies of T cells, helper T cells, and cytotoxic T cells were diminished as compared to the untreated controls (**Figures 6B, D–F**). Analysis of MDSC sub-populations demonstrated that both HIS48^hi^ and HIS48^lo^ MDSCs were significantly elevated on day 7, although the total increase in MDSCs appeared to be driven predominantly by the elevation of the HIS48^lo^ sub-population (**Supplementary Figure 6**).

**Figure 7:**
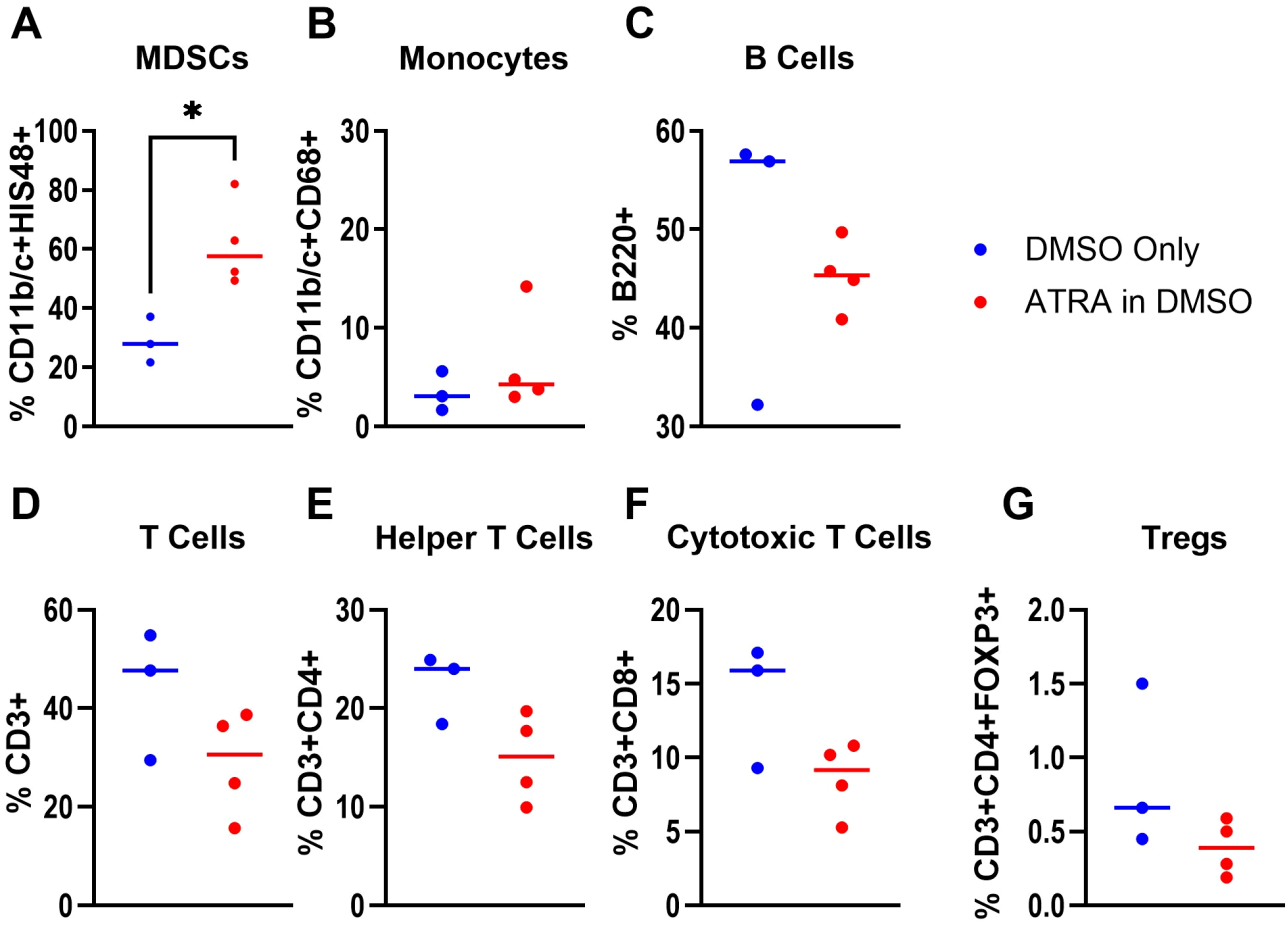
Intraperitoneal delivery of high-dose ATRA in DMSO alters MDSCs as compared to delivery of DMSO alone. **(A)** MDSCS were significantly elevated in uninjured rats injected with ATRA at a dose of 10mg/kg dissolved in DMSO as compared to rats injected with DMSO alone. **(B–G)** Other immune cell populations were not significantly altered. Significance was determined using a two-tailed *t* test for each comparison. *P < 0.05 as indicated.

Lastly, to determine whether these effects were caused by the higher dose of ATRA or by the DMSO in which it was dissolved, an additional experiment was performed wherein a group of naive, uninjured rats received an intraperitoneal injection with either ATRA at a high dose of 10 mg/kg dissolved in DMSO or DMSO alone containing no ATRA. Blood was drawn from both groups 12 hours after injections and immune cells were characterized by flow cytometry. The group that received ATRA in DMSO had significantly higher MDSCs than the group that received DMSO only (**Figure 7A**). Other immune cell populations were not significantly affected (**Figures 7B–F**). Analysis of MDSC sub-populations demonstrated that the increase in total MDSCs observed here was driven by an increase specifically in HIS48^lo^ MDSCs, while HIS48^hi^ MDSCs were unaltered (**Supplementary Figure 7**).

## 4 Discussion

Considerable advances have been made in acute trauma care in recent decades, which have significantly increased the rates of survival and limb salvage. However, this progress has sadly coincided with a resultant increase in late-emerging complications (43), highlighting a need for new and improved treatment strategies. Previous work by our lab has consistently demonstrated a correlation between the systemic immune response to trauma and bone regenerative outcomes. In particular, circulating levels of myeloid-derived suppressor cells (MDSCs) at multiple timepoints post-injury have been found to correlate negatively with endpoint bone volume (7). We also found that a nanoparticle-based immunomodulatory treatment in a rat trauma model led to both an expansion in the total number of circulating MDSCs and poorer bone healing, with levels of MDSCs in the treatment group again correlating negatively to endpoint regenerated bone volume (26). Multiple cytokines known to be expressed by MDSCs such as IL-10 and IP-10 have also been negatively correlated to healing in our previous studies (7, 44). Meanwhile, T cells correlate positively to healing in rat models of trauma (7, 26), and specific T cell subsets are known to promote bone formation and facilitate early fracture healing (24). As such, their suppression by MDSCs may be detrimental to successful bone repair. Altogether, these results demonstrate a myriad of correlations between the systemic immune response to trauma and bone regeneration and highlight MDSCs as a promising potential therapeutic target. Based on our findings, we propose that strategies aimed at combating systemic immunosuppression and restoring homeostasis to the systemic immune environment may yield therapeutic benefits after musculoskeletal trauma and therefore warrant additional study.

To begin to explore such strategies, we performed an initial *in vitro* investigation of several therapeutics that have been previously used to target MDSCs for cancer treatment. Blood immune cells derived from our rat trauma model were treated *in vitro* with all-trans retinoic acid (ATRA) at three different concentrations. ATRA at all three concentrations resulted in a decrease in total MDSCs as compared to untreated controls (**Figure 1A**). The other therapeutics tested *in vitro* did not diminish the relative frequency of MDSCs in this study (**Supplementary Figure 2**). However, these were not exhaustively tested at a variety of concentrations or at later timepoints than 24 hours in culture, so future work would be necessary to determine whether altering these experimental conditions might result in successful MDSC depletion. Furthermore, because our analyses were limited to flow cytometric characterization with a small group of antibodies, it is possible that these drugs modulated the immune cells in ways that were not detectable here. Another interesting finding in this study was that ATRA diminished the relative frequency of MDSCs *in vitro* in blood derived from naive, uninjured rats as well (**Supplementary Figure 3**). Normally, MDSCs in healthy humans and in healthy mice are rarely detected and only in very small numbers among peripheral blood mononuclear cells (45, 46). In uninjured rats, however, cells expressing the recognized rat markers for MDSCs comprise a substantial portion of the immune cells in the blood. Markers for MDSCs notably differ among species, with the granulocytic marker HIS48 being used to identify MDSCs in rats while Gr-1 is used in mice and various other markers are used in humans (47). Therefore, whether there are inherent differences in the relative frequencies of MDSCs between species or whether any observed differences are instead a result of the different markers used for characterizing the cells remains unclear. Nonetheless, the observation that ATRA diminished MDSCs derived from the naive rats as well as those derived from the trauma rats showed initial promise for its therapeutic potential in our model.

Interestingly, a significant decrease in monocytes was also observed for two of the ATRA concentrations tested *in vitro* in blood derived from the trauma model (**Figure 1B**). This effect was unexpected, as ATRA has been shown to increase differentiation of MDSCs into mature myeloid cell types including monocytes (32). ATRA has been shown in some models to exert anti-inflammatory effects on monocytes and macrophages, decreasing toll-like receptor 2 expression, downregulating the production of proinflammatory cytokines by monocytes, and shifting macrophages from the M1 pro-inflammatory phenotype to the M2 anti-inflammatory or pro-regenerative phenotype (48). A decrease in the relative frequency of monocytes was unexpected however. It is also worth noting that circulating monocytes in our rat model of musculoskeletal trauma have also been previously correlated to poor bone regeneration, so a decrease in both populations could still yield therapeutic benefits (7).

Given these positive *in vitro* results, we next aimed to investigate the effects of ATRA on immune cells in our rat model of composite bone and muscle trauma *in vivo*. Following injury, both the ATRA-treated and untreated groups exhibited altered levels of circulating immune cells compared to naive controls that were indicative of trauma-induced immune dysregulation and immunosuppression (**Figure 2**). Higher levels of MDSCs and diminished levels of T cells and T cell subsets were observed in both groups at day 4 and week 1 post-injury before returning to baseline values by week 2, similar to previous results using a rat trauma model (26). Monocytes were also elevated in one of the trauma groups only at week 1 and not at day 4, suggesting a delayed influence of injury on this immune cell population.

Comparing the two trauma groups, daily intraperitoneal injections with ATRA between days 3 and 7 post-injury did not result in a decrease in total blood MDSCs, contrary to the results observed *in vitro*. Notably, however, a significant decrease in one sub-population of MDSCs was observed in the blood at day 4 post-injury, 24 hours after the first ATRA injections (**Figure 3A**). MDSCs are most commonly identified in rats as expressing both granulocyte (HIS48) and monocyte (CD11b/c) markers, but two distinct subsets of the cells can also be distinguished based on their varying degrees of HIS48 expression, as demonstrated in **Supplementary Figure 1**. These two subsets, which we refer to here as HIS48^hi^ MDSCs and HIS48^lo^ MDSCs, may parallel the two subsets which have previously been distinguished in mice and humans, known as granulocytic and monocytic MDSCs (49). Here, only HIS48^hi^ MDSCs were significantly depleted after ATRA treatment, and only at the first timepoint after injections. Interestingly, a similar result was observed in a previous study in which we attempted to deplete MDSCs in a trauma model using another therapeutic platform called Synthetic Nanoparticle Antibodies (26). These nanoparticles were used to target the calcium binding protein S100A8/A9 which is involved in MDSC signaling and accumulation (50). Synthetic Nanoparticle Antibodies selectively depleted the HIS48^hi^ MDSCs, possibly owing to higher levels of S100A8/A9 expression by this subset. Besides MDSCs, regulatory T cells (Tregs) were also diminished in the ATRA-treated group at both day 4 and day 7 post-injury as compared to the untreated trauma group (**Figure 2G**). Given that Tregs are expanded by MDSCs, this result could have been a secondary effect of the ATRA modulating the function of MDSCs, although additional assays would be necessary to confirm this. Despite a subset of MDSCs and Tregs being diminished, no changes were observed in the relative frequencies of the T cell populations measured after ATRA treatment.

Additionally, ATRA treatment did not improve functional bone regeneration, as no differences were detected in the average bone volume, bone mineral density, torsional strength, or torsional stiffness between the treatment and untreated control groups (**Figure 4**). Given that total MDSCs were not significantly depleted and the HIS48^hi^ MDSC subset was only depleted at one timepoint, it remains unclear whether systemically targeting and successfully depleting MDSCs may yield a therapeutic benefit for bone regeneration. Notably, linear regression analysis revealed a negative correlation between Tregs and endpoint bone volume across all injured rats (**Figure 5**). Although this could be interpreted as evidence that Tregs might have potential as a therapeutic target, the fact that Tregs were diminished in the ATRA-treated group in this study while the group’s bone healing remained unchanged somewhat contradicts this interpretation. Still, it is also possible that Tregs were not diminished significantly enough or for a long enough duration to yield a therapeutic benefit. It is also worth noting that some recent studies have indicated pro-regenerative roles of local Tregs in the healing of bone and other tissues (51, 52), but the relationship between systemic Tregs and healing has not been studied as extensively. Moreover, depletion of immunosuppressive cells such as Tregs and MDSCs could yield other benefits besides improving healing, such as combating infections.

Finally, given the diminished subset of MDSCs and Tregs following ATRA treatment in the previous *in vivo* study, a follow-up study was conducted to assess the effects of a higher dose of ATRA on circulating immune cells. To facilitate administration of a higher ATRA dose, the ATRA was dissolved in DMSO rather than in olive oil. Rats with composite bone and muscle trauma were injected with ATRA twice, at days 3 and 7 post-injury, and blood was collected 12 hours after each injection. We chose to collect blood sooner after injections to investigate whether the effects of ATRA were more potent over shorter time horizons. Once again, Tregs were diminished in the ATRA-treated group as compared to the untreated group, while total MDSCs remained unchanged after the first injections (**Figure 6**). Unexpectedly, however, MDSCs were elevated in the ATRA-treated group at day 7, 12 hours after the second round of ATRA injections. It is worth noting that DMSO possesses immunomodulatory properties, with both inflammatory and anti-inflammatory effects of the compound reported in humans and in rodents (53, 54). Therefore, to further investigate whether the observed elevation of MDSCs was due to the higher dose of ATRA or the use of DMSO, a final experiment was conducted using naive rats injected with either ATRA in DMSO or DMSO alone. Here, the ATRA-treated group possessed higher MDSCs 12 hours after injections than the DMSO-only group, suggesting that the high dose of ATRA itself was the likely contributor to the unexpected MDSC elevation observed in the trauma rats (**Figure 7**). It is worth noting the doses of ATRA used in this study were similar to those used previously to successfully suppress MDSCs in mouse cancer models (34, 55), with considerations made for differences in size and metabolic rates between species. This apparent contradiction between our results and previous literature likely underscores the heterogeneity of MDSCs and potential differences in the cells across models and species.

One opportunity for further study is to assess the immunosuppressive capacity of MDSCs before and after treatment with ATRA. Previous studies of ATRA in cancer models have employed T cell proliferation assays and other methods for quantifying the suppressive effects of MDSCs on other immune cells, whereas this study was limited to examining only the relative frequencies of circulating MDSCs and other immune cells by flow cytometry. As such, although total MDSCs were not reduced after *in vivo* ATRA treatment in this study, it is possible that their immunosuppressive effects on other cells were altered in ways that could not be detected using the methods here. Still, specific mechanisms of MDSC-mediated immunosuppression have not been previously correlated to bone healing outcomes, and therefore assays for such mechanisms were somewhat beyond the scope and purposes of this study. Furthermore, the ability of ATRA to diminish the immunosuppressive functions of MDSCs has already been reported extensively in cancer studies.

Another critical area of future work is to develop improved methods for characterizing the highly heterogeneous population of myeloid derived immune cells termed MDSCs, particularly in rats. While extensive research has been performed on MDSCs in mice and in humans, literature on MDSCs in rats is scarce, making broad comparisons between the species difficult. For example, while it has been speculated that the HIS48^hi^ and HIS48^lo^ MDSC populations observed in rats may mirror the granulocytic and monocytic MDSC subsets previously defined in mice and humans, no study to date has provided considerable evidence to link these subsets across the species. Therefore, the discrepancy between the MDSC-modulating effects of ATRA previously observed in studies of humans and mice and the more limited effects of ATRA on MDSCs observed here in this rat model may be due in part to phenotypic differences between the cells across species, making the translation of these results challenging. Additional characterization of rat MDSCs, their phenotypic markers, and their roles in the immune response to injury will be necessary to overcome this challenge.

Certain limitations to this study should be addressed. First, this study included no analysis regarding the healing outcomes of the injured muscle tissue adjacent to the osteotomized bone. Given that this work was motivated largely by previous findings correlating circulating MDSCs with impaired bone healing, the primary purpose of this study was to assess the effects of an immunomodulatory therapeutic strategy on these cells and on subsequent bone regeneration. While the analysis of muscle healing could provide further insight into the effects of ATRA treatment on the overall healing process, such analysis would have required additional treatment groups sacrificed at earlier timepoints, due to the more rapid time course of muscle healing as compared to bone healing. Therefore, evaluation of muscle regeneration was beyond the scope of this work. Another limitation to this study was the use of only female animal subjects. To date, the correlations observed between circulating MDSCs and bone regeneration in this trauma model have been characterized only in female rats. Future work could therefore expand on these findings by investigating sex-related differences in both the systemic immune response to composite trauma and the effects of immunomodulatory therapeutics such as ATRA on the immune system. Finally, it should be acknowledged that bone morphogenetic protein-2 (BMP-2) has been shown to have immunomodulatory effects such as macrophage stimulation and upregulation of cytokines involved in angiogenesis (56). Therefore, all groups of injured rats in our *in vivo* studies were treated with BMP-2 to eliminate the confounding factor of its immunomodulatory effects. The bone defects in this study were critically sized, and therefore a local treatment such as BMP-2 was necessary for any significant regeneration of bone volume to occur. It is unlikely that a systemic immunomodulatory therapeutic strategy alone would result in the healing of a critically-sized bone defect. However, when used in conjunction with a local treatment, we posit that a systemic immunomodulatory treatment might augment the therapeutic benefit of a local treatment, resulting in greater healing. This might, for example, allow smaller effective doses of BMP-2 to be administered while still yielding successful bone regeneration, which could be beneficial given the adverse side effects that have been reported for use of BMP-2, especially at higher doses (57).

In conclusion, this study examined the effects of ATRA on circulating immune cells and bone healing using a rat model of composite bone and muscle trauma. While certain immunomodulatory effects of the treatment were observed, these effects differed between *in vitro* and *in vivo* experiments and were also dose-dependent *in vivo*. Furthermore, improvements in bone healing following ATRA treatment were not observed in this study. Altogether, these results demonstrate some potential for the use of ATRA as an immunomodulatory therapeutic following musculoskeletal trauma, but a challenge remains in achieving successful *in vivo* depletion of total MDSCs, as has been accomplished in mouse cancer models. Further research will be necessary to determine whether this challenge is a result of phenotypic differences between rat MDSCs and MDSCs in other species, or whether this result is unique to MDSCs in the context of trauma. Moreover, it remains unclear whether successful depletion of both sub-populations of MDSCs in the peripheral blood of rats has the potential to improve trauma healing outcomes. Ultimately, further studies are needed to more adequately characterize the role of systemic immune dysregulation in trauma outcomes and to develop immunomodulatory interventions that enhance bone regeneration.

## Data Availability

The original contributions presented in the study are included in the article/**Supplementary Material**. Further inquiries can be directed to the corresponding author.
