# Supplementary material for: Systemic administration of all-trans retinoic acid modulated circulating immune cells but failed to improve healing in a preclinical model of musculoskeletal trauma
Source: Front Immunol. 2026 Jul 28;17:1683993. doi: 10.3389/fimmu.2026.1683993 (PMC13457108; doi:10.3389/fimmu.2026.1683993)
Supplement: Supplementary file 1 [file DataSheet1.pdf]

Supplementary Material

1 Supplementary Tables

Supplementary Table S1. List of therapeutics used *in vitro* for MDSC depletion.

Therapeutic	Concentration	Supplier	Reference
All-Trans Retinoic Acid	0.2 μM, 2μM, 20 μM	United States Pharmacopeia	1674004
Vitamin E	10 μM	Millipore Sigma	47782
Anti-TSPAN33 Antibody	25 μg/mL	Thermo Fisher Scientific	14329-1-AP
Anti-CXCR2 Antibody	10 μg/mL	R&D Systems	MAB8110-SP

Supplementary Table S2. List of antibodies used for flow cytometry.

Target	Conjugate	Dilution	Supplier	Reference
CD32	None	1:25	BD Biosciences	550271
HIS48	FITC	1:75	Thermo Fisher Scientific	11-0570-82
CD11b/c	PE	1:75	Thermo Fisher Scientific	12-0110-82
B220	PE-Cy7	1:75	Thermo Fisher Scientific	25-0460-82
CD68	Alexa Fluor 647	1:75	Bio-Rad Laboratories	MCA341A647
CD3	PE	1:75	Thermo Fisher Scientific	12-0030-83
CD4	FITC	1:75	Thermo Fisher Scientific	11-0040-82
CD8	Pe-Cy7	1:75	Thermo Fisher Scientific	25-0084-82
FOXP3	APC	1:75	Thermo Fisher Scientific	17-5773-82

2 Supplementary Figures

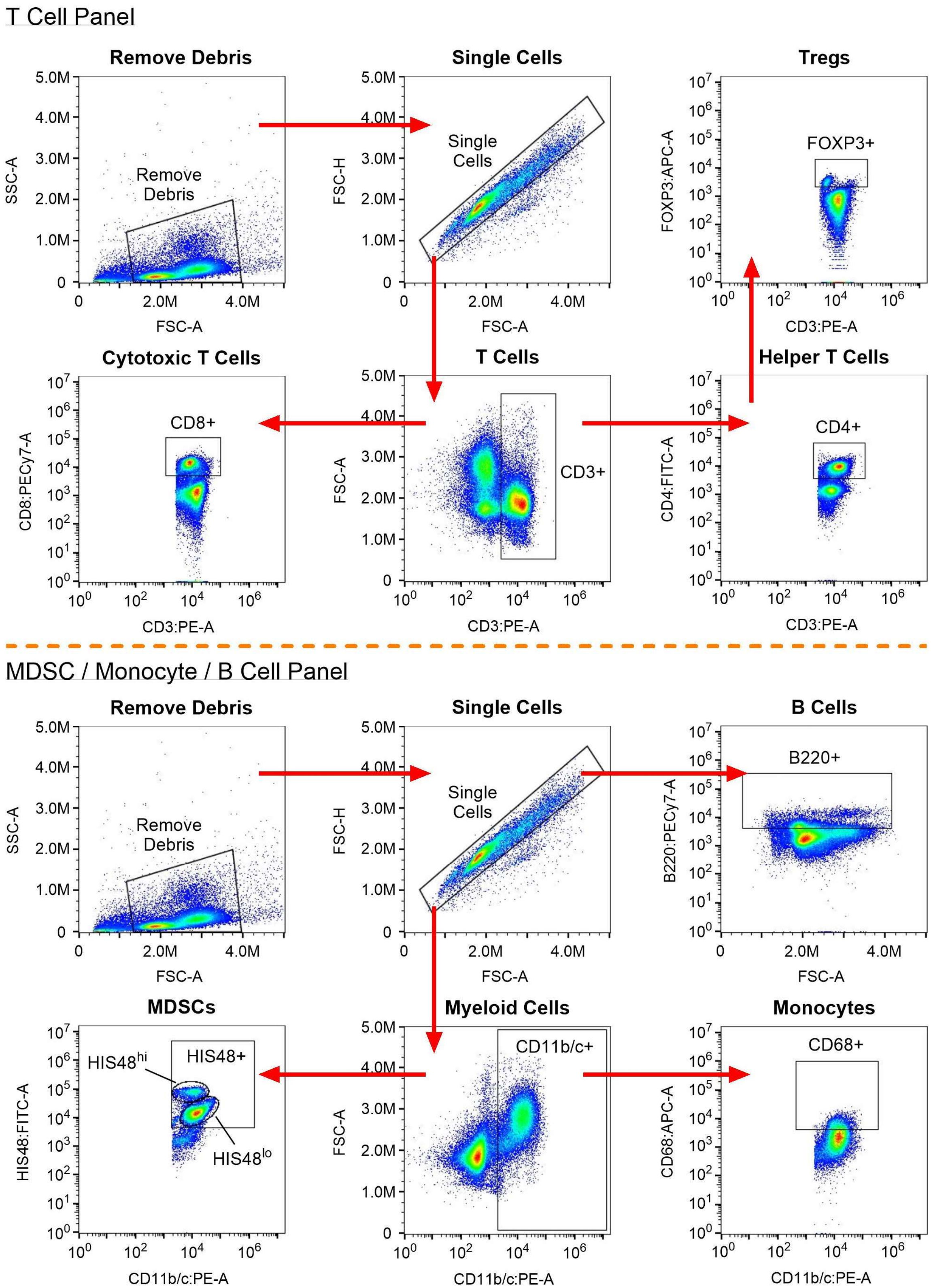

Supplementary Figure S1. Gating strategy for flow cytometry data analysis.

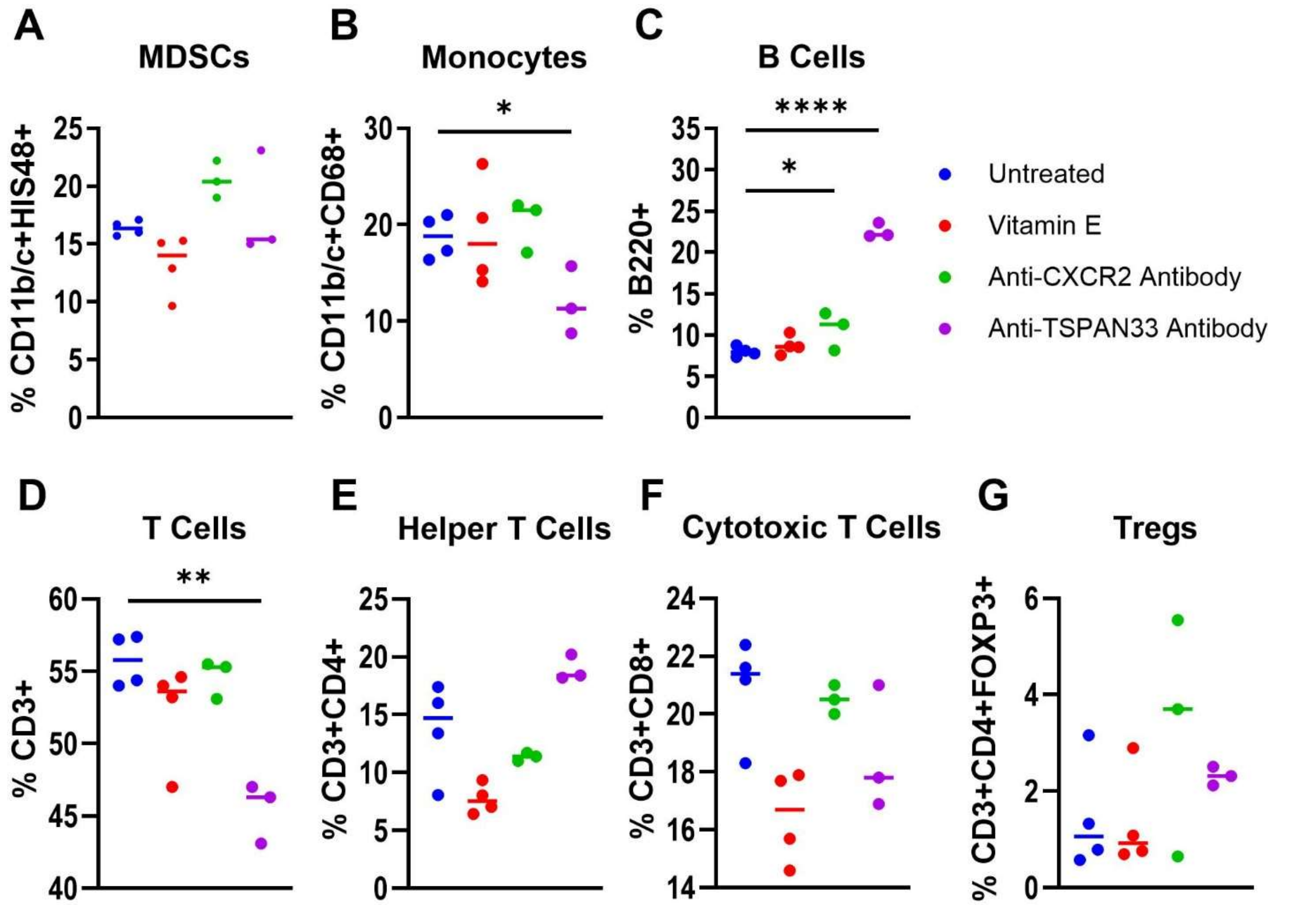

Supplementary Figure S2. Immunomodulatory therapeutics did not alter MDSCs *in vitro* in blood derived from rats with musculoskeletal trauma. (**A**) MDSCs were not affected by *in vitro* treatment with vitamin E, anti-CXCR2 antibodies, or anti-TSPAN33 antibodies. (**B**) Monocytes were depleted following treatment with anti-TSPAN33 antibodies. (**C**) B cells were elevated following treatment with both anti-CXCR2 antibodies and anti-TSPAN33 antibodies. (**D**) T cells were depleted following treatment with anti-TSPAN33 antibodies. (**E–G**) Other immune cell populations were not significantly altered by any of the treatments. Significance was determined using one-way ANOVA with Dunnett's post-hoc test. *P < 0.05; **P < 0.01; ***P < 0.001; ****P < 0.0001 as indicated.

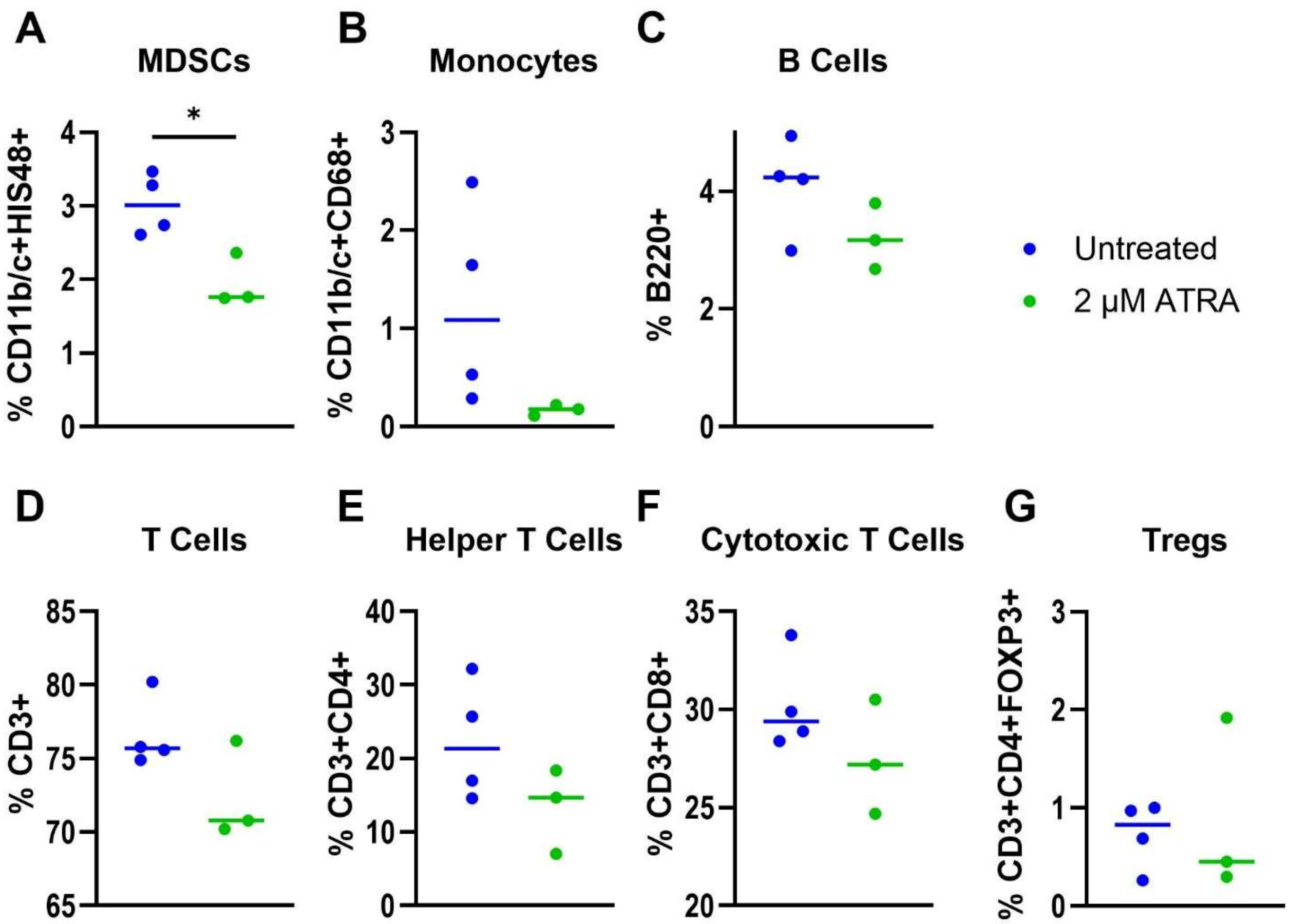

Supplementary Figure S3. ATRA depleted MDSCs *in vitro* in blood derived from naive, uninjured rats. (**A**) MDSCs were significantly depleted following *in vitro* treatment with ATRA at a 2 μM concentration. (**B–G**) Other immune cell populations were not significantly altered. Significance was determined using a two-tailed t test for each comparison. *$P < 0.05$ as indicated.

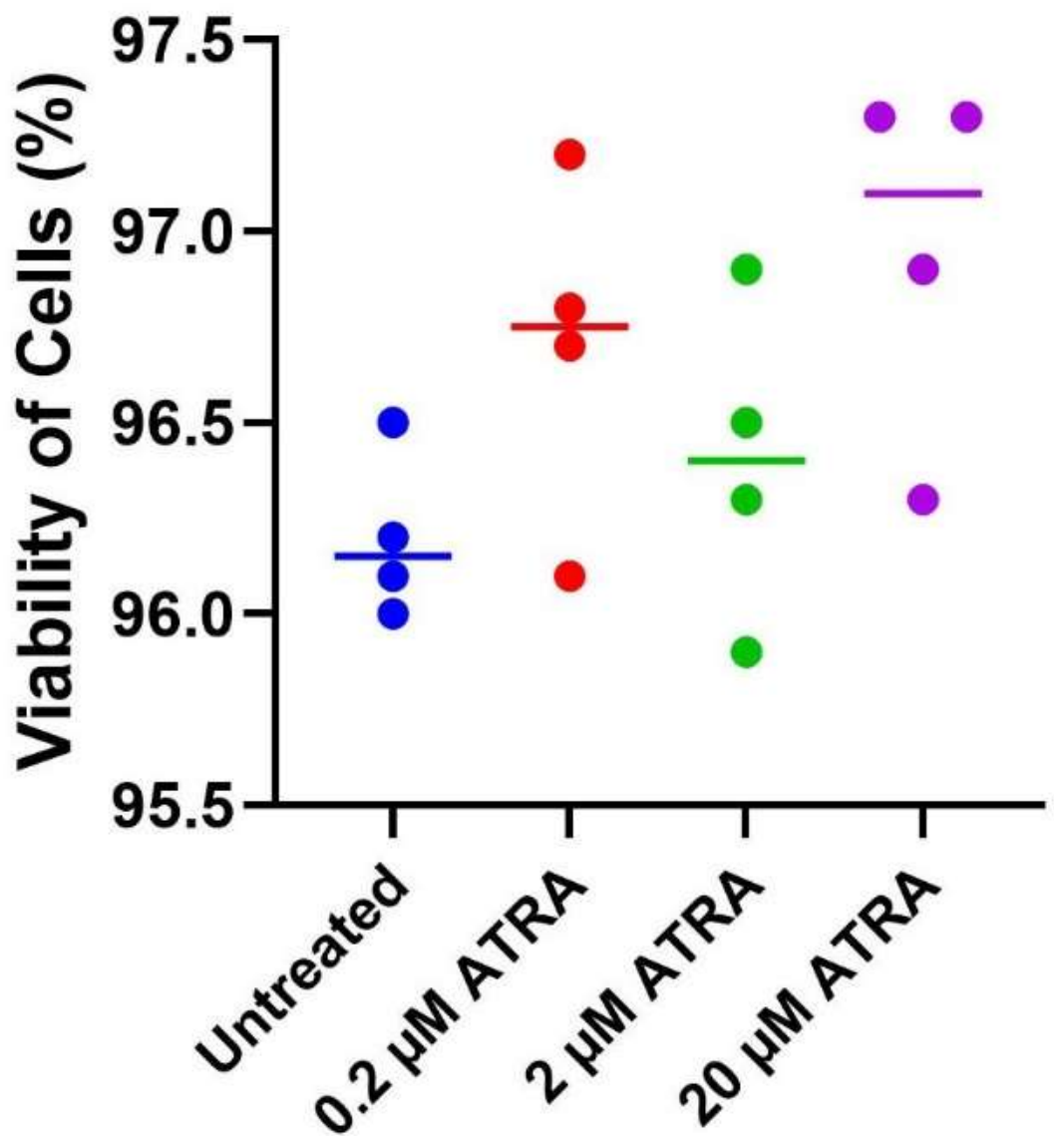

Supplementary Figure S4. Treatment of peripheral blood mononuclear cells with ATRA *in vitro* did not significantly alter cell viability. Cells were incubated either untreated or with ATRA at a concentration of 0.2 µM, 2 µM, or 20 µM. Significance was assessed using one-way ANOVA with Dunnett's post-hoc test.

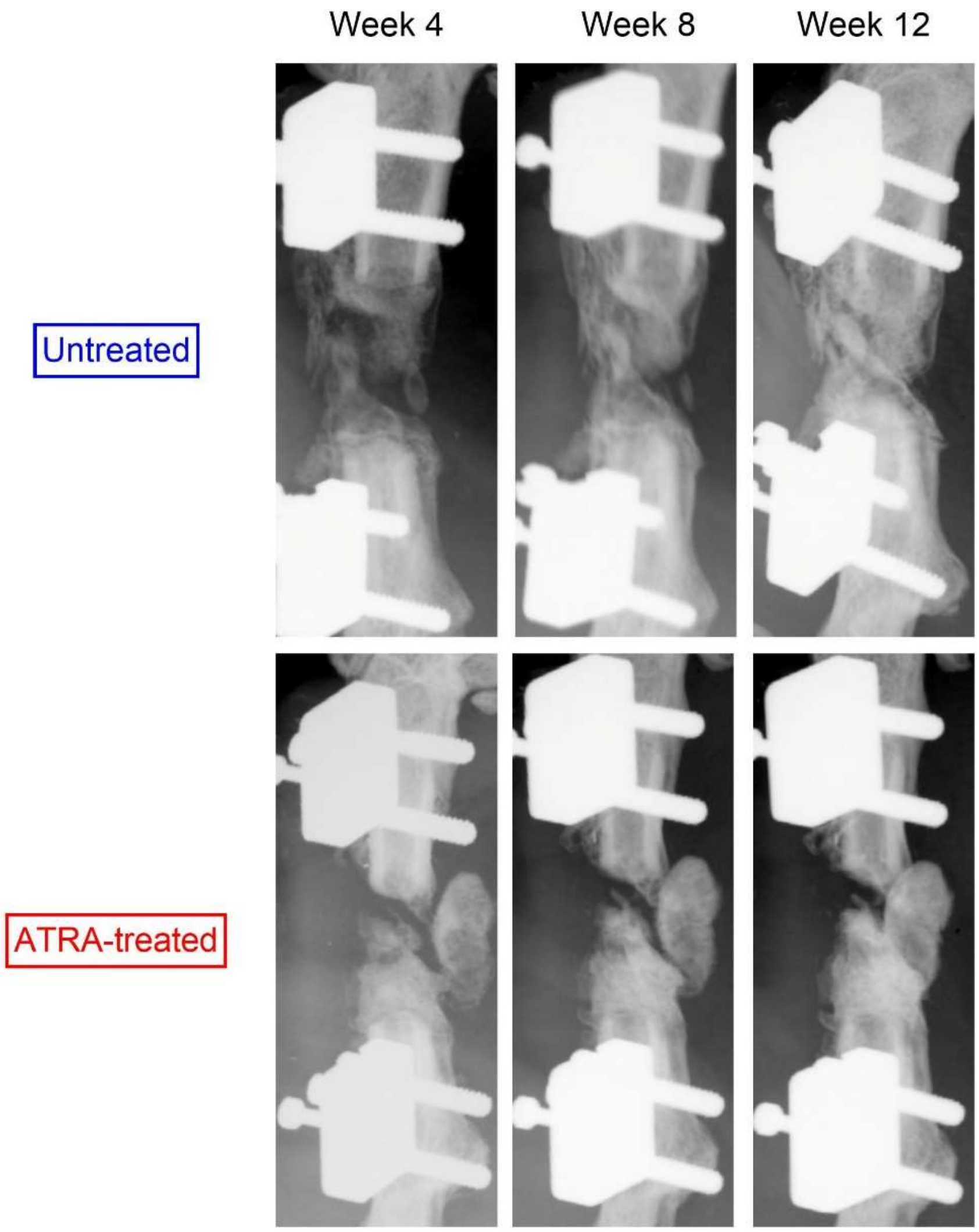

Supplementary Figure S5. Representative radiographs (median healing sample for each treatment group) demonstrating bone formation at weeks 4, 8, and 12 post-injury.

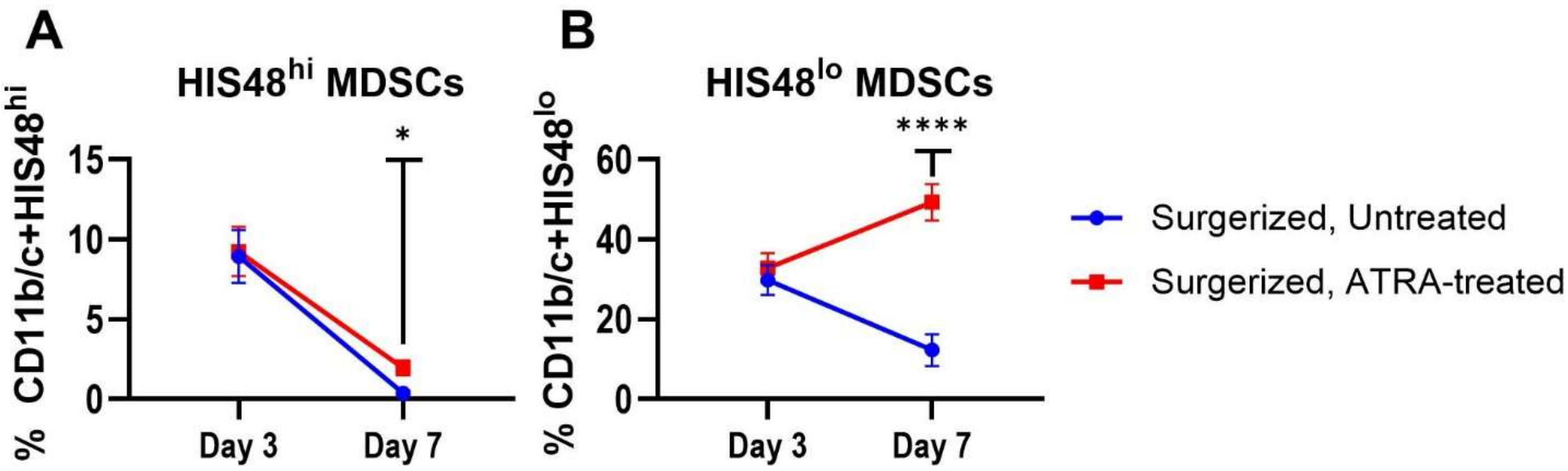

Supplementary Figure S6. MDSC sub-populations were elevated following treatment with ATRA at a higher dose. (**A–B**) HIS48^{hi} MDSCs and HIS48^{lo} MDSCs were not altered at day 3 following a single injection with ATRA at a dose of 5 mg/kg, but they increased following a second ATRA injection at day 7. n = 6 to 7 per group. Significance was determined using a two-tailed t test for each comparison. *P < 0.05; ****P < 0.0001 as indicated.

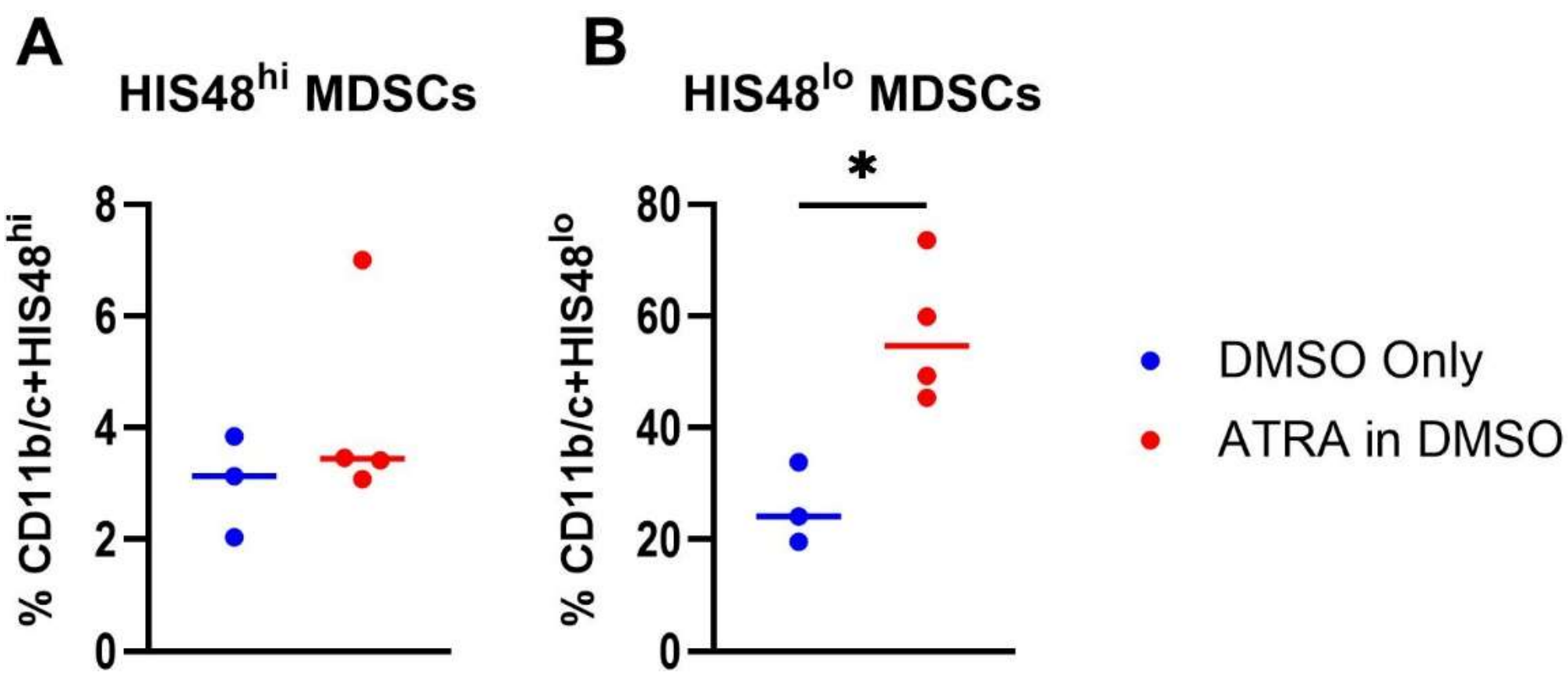

Supplementary Figure S7. Intraperitoneal delivery of high-dose ATRA specifically alters HIS48^{lo} MDSCs in uninjured rats. (**A**) Circulating HIS48^{hi} MDSCs were not altered by treatment with ATRA in DMSO as compared to DMSO alone. (**B**) Circulating HIS48lo MDSCs were elevated following treatment with ATRA in DMSO. Significance was determined using a two-tailed t test for each comparison. *P < 0.05 as indicated.
