# Supplementary material for: Systemic administration of all-trans retinoic acid modulated circulating immune cells but failed to improve healing in a preclinical model of musculoskeletal trauma
Source: Front Immunol. 2026 Jul 28;17:1683993. doi: 10.3389/fimmu.2026.1683993 (PMC13457108; doi:10.3389/fimmu.2026.1683993)
Supplement: Supplementary file 2 [file DataSheet2.pdf]

frontiers

The International Society for the Advancement of Cytometry (ISAC) has highlighted the importance of including comprehensive methodological information to ensure data reproducibility and reliability. In line with this, Frontiers in Immunology now requires authors to submit a checklist for manuscripts that involve flow or mass cytometry. This checklist helps standardize the reporting process, improving the quality and transparency of published data. By doing so, we support scientific progress, making it easier for other researchers to replicate and validate experiments.

This form should be submitted with any manuscripts using flow or mass cytometry.

Sample/specimen/material description

☐ Total blood

☑ PBMCs

☐ Organ digests

Other_______________________________________

Did the samples suffer any treatment before or after incubation with the antibodies?

☑ Drug All-Trans Retinoic Acid

☑ Cell permeabilization Cytofix/Cytoperm buffer (BD Biosciences)

☐ Dye _______________________________________

☐ Propidium iodine

☐ Not applicable

Other _______________________________________

Instrument and antibodies

Name of the Cytometer BD Accuri C6 Plus

Antibodies and targets	Fluorochrome/ Metal	Catalog number/Company
e.g. anti-CD4	FITC	Cat. XXX/ XXX ltd.
CD32	None	550271 / BD Biosciences

HIS48	FITC	11-0570-82 / Thermo Fisher Scientific
CD11b/c	PE	12-0110-82 / Thermo Fisher Scientific
B220	Pe-Cy7	25-0460-82 / Thermo Fisher Scientific
CD68	Alexa Fluor 647	MCA341A647 / Bio-Rad Laboratories
CD3	PE	12-0030-83 / Thermo Fisher Scientific
CD4	FITC	11-0040-82 / Thermo Fisher Scientific
CD8	Pe-Cy7	25-0084-82 / Thermo Fisher Scientific
FOXP3	APC	17-5773-82 / Thermo Fisher Scientific

Data analyses

1. Name of the software FlowJo
2. Reference gating strategy in the manuscript or supplementary material

Gating strategy in (eg Figure X) Supplementary Figure S1
